# Improving the Management of Diabetes in a Secure Forensic Psychiatric Setting in the UK

**DOI:** 10.1192/bjo.2025.10580

**Published:** 2025-06-20

**Authors:** Raymond Effah, Maria Coppel, Christopher Lawrence

**Affiliations:** 1 Portsmouth Hospitals University NHS Trust, Portsmouth, United Kingdom; 2 Hampshire and Isle of Wight Healthcare NHS Foundation Trust, Southampton, United Kingdom

## Abstract

**Aims:** To measure the quality of care given to diabetic patients while admitted to a secure forensic setting. Across psychiatric services, individuals with a serious mental illness have higher rates of diabetes compared with the general population. Patients in forensic inpatient services often have long lengths of stay in hospital which should allow optimisation of diabetes management. We hypothesise that despite intervention, standards in diabetic management are not met.

**Methods:** The first cycle of a retrospective clinical audit was conducted across 5 secure wards at Ravenswood Medium and Low Secure Hospital. Results were collected from paper drug and insulin charts and electronic patient records. Audit standards were set using NICE guideline NG28, European Association for the Study of Diabetes, and Southern Health NHS Foundation Trust Guidelines. The following parameters were audited: Frequency of normal capillary blood sugars (CBG), frequency of blood sugar monitoring, management of hyperglycaemic episodes with short-acting insulin and ketone tests, HbA1c level currently and on admission. The initial audit of September 2024 showed significant underperformance. Following this we implemented feedback sessions of the audit findings with the multidisciplinary team to identify possible areas of improvement; information sessions to highlight monitoring guidelines, and dietary posters for patient areas. January 2025 was then reaudited.

**Results:** Of the original 18 patients, 2 had been discharged, and 16 were identified for the re-audit. Non-compliance with diabetic monitoring improved from 19% to 9%. The average number of attempted CBG measurements per day was 1.32 (previously 1.17, audit standard 2.31). On average, 31% of the CBG readings were in the target range (down from 39%, audit standard 70%). Following an episode of hyperglycaemia, novorapid was administered 68% of the time (previously 67%) and a ketone test was offered 37% of the time (previously 7%, audit standard 100%). Between audit cycles, 56% of patients had their diabetic care plan reviewed by a doctor and 25% of cases were discussed with diabetes specialist services.

**Conclusion:** Our targeted interventions have improved the frequency of monitoring and reduced patient non-compliance to monitoring in a secure setting. However, diabetic control has worsened, highlighting the complexity of the management of diabetes in this patient population. Increased monitoring may have identified poor diabetic control, highlighting room for further improvement. We propose that improved access to specialist diabetes advice and availability of healthier dietary choices for patients could improve diabetic management in the future.

